# A Framework for Text Classification Using Evolutionary Contiguous Convolutional Neural Network and Swarm Based Deep Neural Network

**DOI:** 10.3389/fncom.2022.900885

**Published:** 2022-06-29

**Authors:** Sunil Kumar Prabhakar, Harikumar Rajaguru, Kwangsub So, Dong-Ok Won

**Affiliations:** ^1^Department of Artificial Intelligence Convergence, Hallym University, Chuncheon, South Korea; ^2^Department of ECE, Bannari Amman Institute of Technology, Sathyamangalam, India

**Keywords:** natural language processing, Differential Evolution, Particle Swarm Optimization, Convolutional Neural Network, deep neural network

## Abstract

To classify the texts accurately, many machine learning techniques have been utilized in the field of Natural Language Processing (NLP). For many pattern classification applications, great success has been obtained when implemented with deep learning models rather than using ordinary machine learning techniques. Understanding the complex models and their respective relationships within the data determines the success of such deep learning techniques. But analyzing the suitable deep learning methods, techniques, and architectures for text classification is a huge challenge for researchers. In this work, a Contiguous Convolutional Neural Network (CCNN) based on Differential Evolution (DE) is initially proposed and named as Evolutionary Contiguous Convolutional Neural Network (ECCNN) where the data instances of the input point are considered along with the contiguous data points in the dataset so that a deeper understanding is provided for the classification of the respective input, thereby boosting the performance of the deep learning model. Secondly, a swarm-based Deep Neural Network (DNN) utilizing Particle Swarm Optimization (PSO) with DNN is proposed for the classification of text, and it is named Swarm DNN. This model is validated on two datasets and the best results are obtained when implemented with the Swarm DNN model as it produced a high classification accuracy of 97.32% when tested on the BBC newsgroup text dataset and 87.99% when tested on 20 newsgroup text datasets. Similarly, when implemented with the ECCNN model, it produced a high classification accuracy of 97.11% when tested on the BBC newsgroup text dataset and 88.76% when tested on 20 newsgroup text datasets.

## Introduction

The amount of data to be processed is too large with the advances in the development of information technology (Zheng et al., 2016). Extracting useful information from a huge amount of data is quite important as it saves a lot of time and effort. Therefore, in the field of engineering and technology, effective ways to extract useful information by filtering useless information is an important area of research (Johnson and Zhang, 2015). The representation of information is also pretty diversified including sound, text, images, etc. as there is a rapid rise in the enormous amount of data generated (McCallum, 1998). Fewer network resources are required by text when compared with sound and images, and it is easy for upload and download purposes. Text is always a vital carrier of information as even other forms of data can be represented by it. Achieving the fruitful results of text processing is highly time-consuming and therefore obtaining the data and processing it with effective algorithms can pave a way for good results (Conneau et al., 2017). Therefore, to achieve a free human-machine interaction, text classification becomes a quite interesting and critical technology in the field of Artificial Intelligence (AI). The main intention of text classification is to assign a huge quantity of text to one or more required categories depending on the content and attributes of the documents (Santos and Gatti, 2014). Rule-based and statistical classification-based methods are two categories of text classification (Onan, 2016). More knowledge and rule bases are required in the rule-based classification techniques. The rule development and the subsequent process of updating them make it quite difficult and suitable for application in a specific field. Depending on the statistical knowledge, these statistical learning methods are present, and they establish the learning parameters of the respective data model by means of applying sample statistics and then on the training set it is calculated and subsequently the training of the classifiers is conducted. Leveraging text classification methods is quite an important aspect in the field of Natural Language Processing (NLP) and text mining. For the text classification system, a large number of statistical machine learning techniques are implemented (Chen K. et al., 2016). In many real-time applications, text classification problems have been widely studied and represented in literature (Altinel and Ganiz, 2018). As far as the text classification and categorization of documents are considered, it is usually split into feature extraction, dimension mitigation, selection of classifiers, and evaluations. There are four various levels of the scope of text classification systems, namely document level, paragraph level, sentence level, and sub-sentence level (Comite et al., 2003). The classification tasks employed for text classification include KNN (Han et al., 2001), SVM (Sun et al., 2009), tree-based classifiers (Murthy, 1998), graph CNN methods (Yao et al., 2019), and other recently proposed deep learning models proposed in Li et al. (2020). Some of the most important and classic works of text classification are reported as follows.

A comprehensive survey of text classification algorithms was elaborated by Aggarwal and Zhai (2012). The advent of machine learning in automated text categorization was proposed in detail by Sebastiani (2002). The proposal of the explicit and implicit syntactic features for text classification (Post and Bergsma, 2013), the bay of tricks for efficient text classification (Joulin et al., 2017), and multilingual text detection with nonlinear neural networks (Li et al., 2015) are some of the trivial works done in the field of text classification. The phrasal and clustered representation was evaluated on a text categorization task in Lewis (1992), and the fast logistic regression for text categorization with variable length N-grams was proposed in Ifrim et al. (2008). DE was used for finely adapting Naïve Bayesian Classifier (NBC) and used for text classification in Diab and El Hindi (2017). A multiple partially observed view for multilingual text categorization (Amini et al., 2009), an iterative deep neighborhood model for text classification (Liu et al., 2020), integrating bidirectional Long Term Short Memory (LSTM) with 2D max pooling for text classification (Zhou et al., 2016), Recurrent Neural Network (RNN) for text classification with multi-task learning (Liu et al., 2016), Recurrent CNN for text classification (Lai et al., 2015), and a character level convolutional network for text classification (Zhang et al., 2015) are some of the most famous deep learning works proposed in the literature. A ranking based deep learning representation for efficient text classification (Zheng et al., 2018), a hierarchical neural network document representation approach for text classification using three different models (Kowsari et al., 2017), a C-LSTM neural network for text classification (Zhou, 2015), and a neural attention model for leveraging contextual sentences for text classification (Yan, 2019) are again some of the works which help the research community to a great extent for further analysis. Based on the recognition of semantic topics, the Chinese texts were classified by Chen Y. W. et al. (2016). An iterative dual attention network for text sentiment classification (Zhu et al., 2020), an aspect level sentiment classification with an interactive model of target and context (Han et al., 2019), and the sentiment analysis using common sense and content information were proposed in Agarwal et al. (2015). The news text classification technique and simulation depending on the hybrid deep learning model was developed in Sun and Du (2021), and an attention-based BiLSTM fused with CNN and gating mechanism for Chinese long text classification was done in Deng et al. (2021). A heterogeneous classifier ensemble based on deep learning and word embedding for text classification was done in Kilimci and Akyokus (2018), and a hybrid CNN-RNN attention-based model for text classification called CRNN was developed in Guo et al. (2018). Hybrid embedding-based text representation for hierarchical multi-label text classification (Ma et al., 2022), a co-attention network with label embedding for text classification (Liu M. et al., 2021), and a bidirectional gated temporal combination with attention for text classification (Ren et al., 2021) are some of the recent works reported in the field of text classification. The label-based attention for hierarchical multi-label text classification network was developed in Zhang et al. (2022) and the hybrid optimization algorithms with feature selection were utilized for text classification in Thirumoorthy and Muneeswaran (2021). As far as security aspects in text classification are concerned, for LSTM-based text classification, the backdoor attacks were mitigated with the help of backdoor keyword identification as implemented in Chen and Dai (2021).

In this work, two deep learning techniques are proposed with the help of swarm intelligence techniques like DE and PSO, and it has been incorporated with CNN and DNN, and finally, the results have been analyzed. DE is a very famous population-based evolutionary algorithm that is utilized for solving multi-dimensional global optimization problems over continuous spaces; thus, in this paper, it has been combined with deep learning for the purpose of text classification. The main intention to use PSO in this work is because of its simple concept, computational efficiency, easy implementation, and robustness to control parameters. Thus, considering these factors, it has been implemented with deep learning for the purpose of text classification in this paper. A few relevant works involving PSO and DE with deep learning for text classification and other important tasks are discussed as follows. The application of PSO for hyper-parameter selection in deep neural networks was done elaborately by Lorenzo et al. (2017a). The hyper-parameter selection in deep neural networks using Parallel PSO too was discussed by Lorenzo et al. (2017b). A PSO based deep learning model for vehicle classification (Alhudhaif, 2022), image classification (Junior and Yen, 2019), hyper spectral image classification (Liu X. et al., 2021), and flash flood detection from satellite images (Tuyen et al., 2021) too was reported in the literature. A text feature selection using the PSO algorithm was implemented by Zahran and Kanaan (2009). A feature selection empowered by self-inertia weight adaptive PSO for text classification was reported by Asif et al. (2022). An enhanced textual data classification using the PSO algorithm was reported by Aro et al. (2020), and the application of DE for neural networks optimization was performed by Baioletti et al. (2020). The feature selection for text and image data using DE with support vector machines and Naïve Bayesian classifiers was done by Dixit and Bansal (2020), and DE was used for fine tuning Naïve Bayesian classifiers with its applications for text classification implemented by Diab and El Hindi (2017). The DE based hyperparameters tuned deep learning models for disease diagnosis and classification were done in Kaliyapillai and Krishnamurthy (2020). The DE based feature selection and classifier ensemble for named entity recognition was done by Sikdar et al. (2012) and evolutionary optimization of ensemble learning to determine sentiment polarity in an unbalanced multiclass corpus was done by Gracia-mendoza et al. (2020). The organization of the paper is as follows. The contribution of the first proposed framework is given in Section First Proposed Framework: ECCNN. and the contribution of the second proposed framework is given in Section Proposed framework 2: Swarm DNN. The results and discussion are elaborated in Section Results and Discussion followed by the conclusion in Section Conclusion and Future Work.

## First Proposed Framework: ECCNN

The proposed framework utilizes the idea of both DE and CNN and is explained as follows.

### Differential Evolution

One of the most famous evolutionary algorithms used widely is DE (Mohamed, 2015). For various optimization tasks such as image processing, signal processing, wireless networking, computer vision, semantic classification, etc., it has been used widely. The training parameters are initialized by DE such as population size *S*, individual dimension *S*_*par*_, crossover probability *CR*, and a mutation scaling parameter *M*. In the starting state, the generation of a population *P*of size *S*and dimension *S*_*par*_ is done utilizing the following equation:


(1) $$\begin{array}{l} p_{i}=Low_{i}+rand\left(S,S_{par}\right)*\left(Upp_{i}-Low_{i}\right),\\ p_{i}\in P,i=1,2,\ldots ,S \end{array}$$

where *Low* represents the lower frontiers of the search space and *Upp*represents the upper frontiers of the search space. In a particular interval [0,1], a random matrix is initiated, and the function utilized is *rand* (., .). A novel and contemporary individual *w*_*i*_ is created from the present parent individual *p*_*i*_ by means of using a mutation operator. The mutation operation is performed by the DE scheme as defined in Equation (2).


(2) $$\begin{array}{l} w_{i}^{t}=p_{r_{1}}^{t}+M*\left(p_{r2}^{t}-p_{r3}^{t}\right) \end{array}$$

where *p*_*r*1_, *p*_*r*2_ and *p*_*r*3_ are different individuals which are chosen randomly for the population at an iteration *t*. An offspring individual from *w*_*i*_ and *p*_*i*_ can be generated by the crossover operator as follows:


(3) $$y_{ij}^{t}=\{\begin{array}{cc} w_{ij}^{t},if & \gamma_{j}\leq CR\begin{array}{cc} or & \delta_{i} \end{array}\\ p_{ij}^{t}, & otherwise \end{array}$$

where the arbitrary value picked for the *j*^*th*^decision variable is represented as γ_*j*_. A random version variable index picked from [1, *S*_*par*_] is represented as δ_*i*_. The fitness functions *fit*_*p*_*i*__,*fit*_*y*_*i*__ of both the parent individual and the offspring *p*_*i*_ and *y*_*i*_, respectively, are done separately.

The best individual for both the parent individual *p*_*i*_ and the offspring *y*_*i*_ is selected based on the computation of the fitness function values and is represented as per the following equation:


(4) $$p_{i}^{t+1}=\{\begin{array}{cc} y_{i}^{t},\;if & fit_{y_{i}}\leq fit_{p_{i}},\\ p_{i}^{t}, & otherwise \end{array}$$

Unless the stop condition is satisfied, the repetition of the previous steps is done. The best individual is returned, and the DE is stopped if the stop condition is satisfied, or else from the mutation phase, it can continue to start again. To perform a mutation, various strategies can be utilized by the DE algorithm where the capability of the search space in terms of exploration can be improved. By representing it “DE/c/d”, the distinguishment between strategies can be done, where the solution to be mutated is denoted by ‘c’ and the number of varied solutions utilized is represented by ‘d’. Two strategies are utilized in this work, where the first one is represented as “DE/best/1” and is represented as:


(5) $$\begin{array}{l} w_{i}^{t}=p_{d}^{t}+M*\left(p_{r2}^{t}-p_{r3}^{t}\right), \end{array}$$

where the second one is represented as “DE/best/2” and is represented as:


(6) $$\begin{array}{l} w_{i}^{t}=p_{d}^{t}+M*\left(p_{r2}^{t}-p_{r3}^{t}\right)+M*\left(p_{r3}^{t}-p_{r4}^{t}\right) \end{array}$$

where the best solution at iteration *t* is indicated by $p_{d}^{t}$

### Proposed Deep Learning Model ECCNN

The deep learning model proposed is that it considers the input instances of a particular data point and at the same time it considers the classification map of contiguous data points too as the input. The inputs of the model are introduced followed by the model construction and the technique required to learn the model parameters are then explained.

#### Inputs to the Model

It is considered that a training set of *n* datapoints is present and a multiclass classification issue of *l* classes is present. The consideration of the training set is done as ${\left\{\left(P_{i},q_{i}\right)\right\}}_{i=1}^{n}$, where the input data which presents the *i*^*th*^ data points are represented as *P*_*i*_ and the class label vector of the *i*^*th*^ data point is represented as $q_{i}={\left[q_{i1},\ldots ,q_{iL}\right]}^{T}\in {\left\{1,0\right\}}^{L}$. *q*_*il*_ = 1 if *P*_*i*_ belongs to the *l*^*th*^class and it is zero otherwise. In the training set *P*_*i*_, in order to classify one data point, two types of data are included by the input of the model as follows:

##### Instance Arrangement

The instances of the data point itself are the first type of input and are considered as follows:*P*_*i*_ = (*p*_*i*1_, …, *p*_*i*|_*P*__*i*_|_), where the length of the sequence is expressed as |*P*_*i*_| and the feature vector of the *k*^*th*^ instance of the *i*^*th*^ data point is represented as *p*_*ik*_.

##### Contiguous Classification Map

The neighborhood of *P*_*i*_ is the second type of input and the classification map of the neighborhood. The neighborhood dataset of *P*_*i*_ is specified as *N*_*i*_. The classification response is initially computed for every class and then a class wise max-pooling operation is applied. The *L* maximum responses for *L* classes are finally concatenated to obtain the classification map of *N*_*i*_. With respect to the *l*^*th*^ class, the classification response of a data point *x*_*j*_ ∈ *N*_*i*_ is denoted as *x*_*jl*_ ∈ [0, 1], and the classification map of *N*_*i*_ is expressed as follows:


(7) $$\begin{array}{l} x_{i}={\left[\underset{j:P_{j}\in N_{i}}{\max}x_{j1},\ldots ,\underset{j:P_{j}\in N_{i}}{\max}x_{jL}\right]}^{T}\in {\left[0,1\right]}^{L} \end{array}$$

where max_*j*_:_*P*_*j*_∈*N*_*i*__*x*_*jl*_ denote the max pooling outcome of the classification acknowledgment over *N*_*i*_ with respect to the *l*^*th*^class. The computation of the classification response *x*_*jl*_ is expressed in the following subsections.

Based on the above explanations, for every input data point *P*_*i*_, 2 inputs are expressed as (*p*_*i*1_, …, *p*_*i*|_*P*__*i*_|_) and *x*_*i*_.

#### Structure of the Model

**Figure 1:**
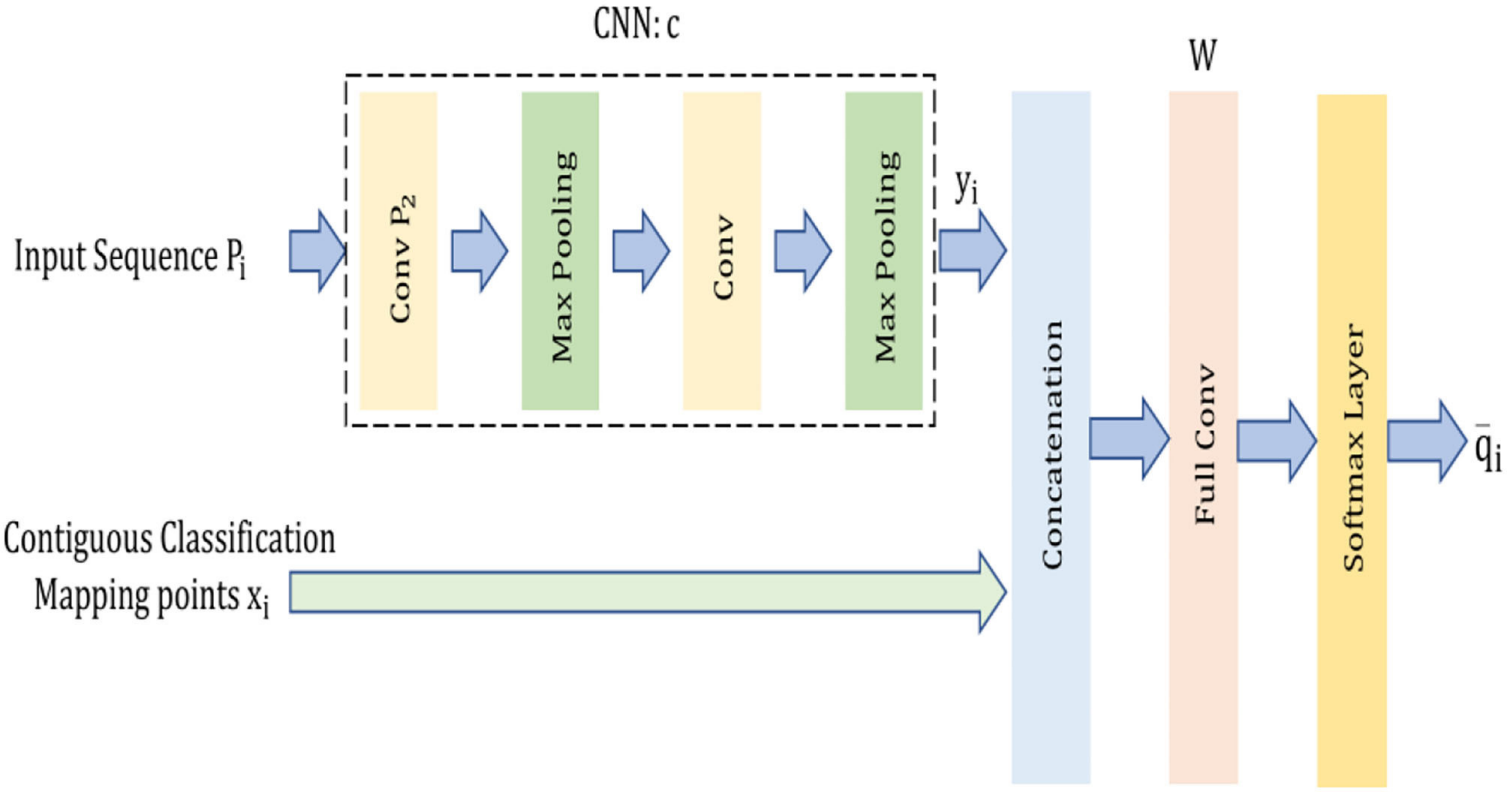
Overview framework of the CCNN model.

Figure 1 illustrates the overview framework of our model. The model comprises a CNN model (Jeong et al., 2020), indicated as *c*, one concatenation layer, 1 Fully Connected (FC) layer, and one Softmax nonlinear transformation layer inspired by the work and architecture in (Liu et al., 2020). The dataflow in the model along with the function of these layers are explained as follows:

(i) The initial transformation of the input sequences of instances to a vector of a d-dimensional vector *y* ∈ ℜ^*d*^ is done by the CNN model ′*c*′which has 3 convolutional layers and 2 max-pooling layers represented as:
  (8) $$\begin{array}{l} y_{i}\in f\left(P_{i}\right) \end{array}$$
(ii) The concatenation of *y*_*i*_ is done by utilizing the neighborhood classification map vector $x_{i}\in {ℜ}^{L}$ by using the concatenation layer. The indication of the concatenated vector is done as follows:
  (9) $$\begin{array}{l} \left[\begin{array}{c} y_{i}\\ x_{i} \end{array}\right]\in {ℜ}^{d+L} \end{array}$$
(iii) The fully connected layer enables the concatenated vector to further mitigate it to an L-dimensional vector and its concatenation weight matrix is expressed as
  (10) $$\begin{array}{l} W=\left[w_{1},\ldots ,w_{L}\right]\in {ℜ}^{d+L}\times L \end{array}$$
  where *w*_*l*_ represents the *l*^*th*^ column which corresponds to the *l*^*th*^ class. The calculation of the output of the FC layer is expressed as
  (11) $$\begin{array}{l} W^{T}\left[\begin{array}{c} y_{i}\\ x_{i} \end{array}\right]={\left[W^{T}\left[\begin{array}{c} y_{i}\\ x_{i} \end{array}\right],\ldots ,W_{L}^{T}\left[\begin{array}{c} y_{i}\\ x_{i} \end{array}\right]\right]}^{T} \end{array}$$
(iv) The Softmax activation layer is used to enable the outputs of the FC layer to normalize the probabilities on the *L* classes and the calculation is done as follows:
  (12) $$\begin{array}{l} \bar{q_{i}}={\left[x_{i1},\ldots ,p_{iL}\right]}^{T}\in {\left[0,1\;\right]}^{L} \end{array}$$
  where,
  (13) $$\begin{array}{l} x_{il}=\frac{\exp\left(w_{1}^{T}\left[\;\begin{array}{c} y_{i}\;\\ x_{i} \end{array}\right]\right)}{\sum_{l^{\prime }=1}^{L}\exp\left(w_{l^{\prime }}^{T}\left[\;\begin{array}{c} y_{i}\;\\ x_{i} \end{array}\right]\;\right)} \end{array}$$
  denote the probability of *P*_*i*_ which belongs to the *l*^*th*^ class. The $\bar{q_{i}}$ is conveyed as the model output vector. The class with the largest probability is chosen in order to determine the class of the designated data point *P*_*i*_ and is expressed as
  (14) $$\begin{array}{l} q^{*}=\arg\underset{l=1,\ldots ,L}{\max\;}x_{il} \end{array}$$

#### Parameter Learning of the Model

The parameter of the CNN model ′*c*′, the contiguous classification mapping points *x*_*i*_, and the connection weight matrix are the three groups of parameters in our proposed model. A unified learning framework is built in order to learn the parameters, so the training data is fit well. By means of using cross-entropy loss functions, the classification error is measured, and by using the squared *l*_2_ norm of the parameters, the model complexity is analyzed in this specific learning framework. To mitigate the overfitting risk, minimizing the classification error is proposed so that the classification performance can be improved, and the complexity of the proposed model can be reduced.

The modeling of the learning problem is done as a minimization problem and the problem objective is expressed as follows:


(15) $$\begin{array}{l} obj\left(W,c\right)=\overset{n}{\underset{i=1}{\sum }}l\left(q_{i},\bar{q_{i}}\right)+\frac{T_{1}}{2}\left(||W|{|}_{2}^{2}+||c|{|}_{2}^{2}\right) \end{array}$$

where $l\left(q_{i},\bar{q_{i}}\right)=-\overset{L}{\underset{l=1}{\sum }}q_{il}\log\left({\bar{q}}_{il}\right)$denote the cross-entropy loss function for the *i*^*th*^ data point. The squared *l*_2_ norm of *W*is represented as $||W|{|}_{2}^{2}$. The squared *l*_2_ norm of the filters of the CNN model *c* is represented as $||f|{|}_{2}^{2}$ and *T*_1_ denotes the tradeoff parameters of the *l*_2_ norm regularization term and the classification error term. To grasp the quintessential parameters and c^*^across the training set, the minimization problem is expressed as follows:


(16) $$\begin{array}{l} \begin{array}{c} W^{*},c^{*}=\arg\underset{W,c}{\min}O\left(W,c\right),\\ s.t.y_{i}=f\left(P_{i}\right),\forall_{i}=1,\ldots ,n \end{array} \end{array}$$

In the learning problem, the convolutional specification vector of the CNN model *y*_*i*_ is introduced for every position of the data and it is inflicted to be identical to the CNN model output, *c*(*P*_*i*_).

#### Optimization Problem

Solving the problem in Equation (16) directly is quite hectic and the classification map *x*_*i*_ is fundamentally a function of *W, c* and *x*_*j*:*P*_*j*_∈*N*_*i*__. Also, there is a coupling of parameters *W*and *c*. Therefore, to solve these problems, the famous Expectation Maximization (EM) algorithm is utilized in this work (Wang et al., 2004). In an alternate manner, updating the parameter specification and the contiguous classification map vectors for every data point is done in an iterative algorithm. The classification map vectors $x_{i}{|}_{i=1}^{n}$ are fixed in M-step and then by minimizing the objective, *W*and *c* are updated. The fixing of the parameters *W*and *c* are done in the E-step so that the neighborhood and the contiguous classification map vectors are updated.

### Proposed Framework Implementation of the ECCNN

The proposed ECCNN framework dependent on the DE algorithm for the enhancement of CCNN is explained in detail. To estimate the optimal architectures and parameters for a CNN, ECCNN is proposed so that the performance of classification is enhanced. The best configuration is searched by DE from a group of parameters and is utilized to assemble and train a CNN. The surviving CNN architectures which are utilized for the classification of text in the form of a word, sentence, etc., usually have just 1D convolution and pooling in most cases; however, 2D convolution operators are implemented in ECCNN. The text from the dataset is provided as an input to the CNN in the form of a word insertion matrix with two dimensions. Therefore, the representation of every word is done by the vector extricated from a word insertion or fixing which is pretrained. To inhibit destroying the word embedding structure, the extraction of features is aided by the 2D convolution operators. The ECCNN consists of three stages, namely starting stage, evaluation stage, and updating stage. A random population *P*with size *S*and dimension *S*_*par*_is created in the starting stage by ECCNN. The total number of hyperparameters utilized to dominate and regulate the CNN configuration is represented as *S*_*par*_. The CNN configuration is nothing but the convolution filters, dropout rate, filter size, convolution filter size, the total number of filters, and the number of neurons in the FC layer. A random value is selected and utilized to start every solution *P*_*i*_, (*i* = 1, 2, …, *S*) and various values are contained in every parameter in *P*.

**Figure 2:**
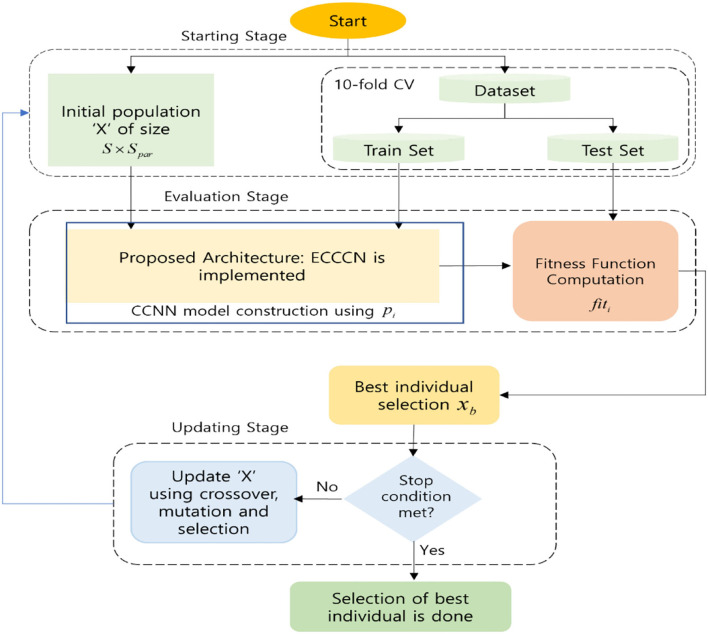
Framework of the proposed ECCNN.

The division of the dataset into training/testing with a 10-fold method is utilized by the two fitness evaluation techniques. Secondly, based on the present *P*_*i*_, the CNN is built by the evaluation stage, where the determination of the number of parallel convolution layers is done by the number of convolution filters. To a parallel convolution layer, the convolution filter size is assigned, and it is followed by a max-over time pooling layer, which helps to mitigate the dimensionality and computational cost. The representation of pooling size is done as (max(*L*) − *fy* + 1, 1), where the sequence length is represented by *L* and the filter size which has allocated the previous convolution layer is expressed as *fy*. In order to consolidate the output from every pooling layer, a concatenation operation is performed, and then it is fed to the FC layer. Depending on the corresponding values from *P*_*i*_, a hidden layer followed and accompanied by a drop out functioning is built by the ECCNN. Once the CCNN is built using *P*_*i*_, for the performance enhancement of the CNN model, the testing set is utilized, and it is done by means of utilizing the fitness value function *fit*_*i*_ of the ongoing solution *P*_*i*_. Then the best solution *P*_*best*_ is selected by DE which has the highest fitness values *fit*_*best*_. Lastly, using the updating stage, the updation of the potential and final solutions of the population *P*is obtained utilizing DE algorithm operators which involve cross over, mutation, and selection steps. Unless the stop criterion is met, the repetition and evaluation of updation stages are done. Figure 2 gives the detailed explanation of the proposed model. The following section gives more comprehensive details about it.

#### Initializing Stage

The generation of the catalog of values that corresponds to every parameter is done. Moreover, the setting of the parameters of the DE algorithm is also done. Choosing the size of the solutions *S* along the utmost number of iterations *t*_max_ is also given a higher significance in this process. The generation of a random integer population *P* is expressed in the following equation with reference to a particular size and dimension *S*_*par*_ as follows:


(17) $$\begin{array}{l} p_{ij}=low_{j}+rand*\left(upp_{j}-low_{j}\right),\\ \;j=1,2,\ldots ,S_{par},i=1,2,\ldots ,S \end{array}$$

The lower and upper frontiers of the *j*^*th*^parameter of *p*_*i*_ ∈ *P*are represented as *L*_*j*_ and *U*_*j*_, respectively.

About 500 perceptible filters with similar sizes and various initialization values are present as far as the size of the convolution filter is concerned. After every convolution layer, the pooling layer along with the max-pooling operation is accommodated which assumes a similar filter size value so that the computation of the pooling size is done quickly. From the pooling layer, the output feature vectors are assembled along with the contiguous classification mapping points, and then a concatenation operation is implemented so that it can be implemented directly into the FC layer. About 400 neurons are comprised in this FC layer and it is initialized by utilizing the uniform mode. In order to get a high classification accuracy at the end of the model, the Softmax layer is utilized.

#### Evaluating Stage

The construction of the CNN model is done at this stage depending on the parameters of the current solution *p*_*i*_. A 10-fold split technique is utilized to assess the fitness function *fit*_*i*_ for every *P*_*i*_. 90% of training data is selected as the training set and 10% of data is selected as the testing set. For 10 times, this evaluation is repeated and the fitness function value *fit*_*i*_ is nothing but the classification accuracy average over ten times and is represented as


(18) $$\begin{array}{l} fit_{i}=\frac{\sum_{l=1}^{10}acc_{l}}{10} \end{array}$$

where *acc*_*l*_ specifies the classification accuracy for the *l*^*th*^ run.

#### Updating Stage

The determination of the finest solution *P*_*best*_ with the highest fitness function value *fit*_*best*_ is done in this stage. The updation of every solution *P*_*i*_ in the current population *P*is done by means of utilizing the three main operators of the DE algorithm. Unless the stop criterion is satisfied, the repetition of the evaluation stage and the update stages are done continuously. When the maximum number of iterations (*t*_max_) is reached, it implies that the stop condition is achieved.

#### Impact of CNN Parameters on the Architecture

The performance of the simulation results is affected greatly by the parameter tuning for all the deep learning algorithms. The developed ECCNN architecture with suitable parameters is explained as follows. An individual configuration is required in order to parametrize the CNN model. The parameters of the individual structure from two layers, namely the convolution layer and the FC layer, are considered in our experiment. The convolution layers are trained at the starting and in total the coding of five various parameters is done for each individual. For every individual, the optimizer is fixed, and the operation is merged. To enhance the accuracy of the classified sentences to the best of their ability on a test set, the parameter values are also changed. The parameters and their respective values are expressed in Table 1.

**Table 1:** Parameter Values of the proposed ECCNN architecture.

**Parameter**	**Value**
Specifications of Convolution Layer:	
Size of the filters	2 to 10
Number of filters per convolution filter size (NFCS)	50, 100, 150, 200, 250, 300, 350, 400, 450, 500
Specification of Fully Connected Layer:	
Dropout Rate Setting	0.2 to 0.8
Initialization Mode Setting	Uniform, normal, LeCun uniform, He uniform
Neuron Number	50, 100, 200, 250, 300, 350, 400

To perform the convolution operation, the number of filters utilized is in the range of 50 to 500 filters per filter size. The contiguous classification mapping points along with the total number of convolution layers are trained in a collateral manner and are associated with the dimension of filter size. To generate a random filter size list, a random function is incorporated where every filter size has values ranging from 2 to 10. In a list, the highest number of generated filter sizes is restricted to only a few ones. To construct the FC layer, the number of neurons utilized is 50, 100, 200, 250, 300, 350, and 400. For the convolution and FC layer, the activation function utilized is the ReLU function. For the FC layer, various initialization nodes are explored such as normal, He uniform, LeCun uniform, and uniform. The famous regularization technique Dropout rate is adopted to avoid the overfitting of CNN and the dropout rate has different ranges starting from 0.2 to 0.8. To train the CNN, the optimizer utilized is Adam. Zero padding is adopted so that sentences with variable lengths can be handled well. To choose the paradigmatic arrangement for every dataset, a 10-fold cross-validation has been utilized to compute the final classification result. The training of the CNN will be done for the dataset from scratch using the selected arrangement and configuration. Thus, for the proposed ECCNN architecture, after several trials and error methods, the specifications which provided good results are analyzed and provided in Table 2 with the subsequent DE parameters such as population size = 60 or 90; DE strategy = DE/best/1, and DE/best/2; and for all the varying values of *M*and *CR*, the results are tabulated in **Tables 5**–**10**. The population size was set as 60 and 90 after several combinations of trial and error, and it was set to those values as it finally gave a good result.

**Table 2:** Architecture specification implementation for the 2 datasets using ECCNN architecture.

**Dataset utilized**	**Filter size**	**Total number of neurons considered**	**NFCS**	**Initialization mode**	**Dropout rate**
20 Newsgroup	[2,3,5]	300	100	Normal	0.5
BBC news group	[2,3,5]	300	100	Normal	0.4

## Proposed Framework 2: Swarm DNN

The proposed framework makes use of the idea of both PSO and DNN and it is implemented as follows.

### Hyperparameter Selection of DNN

**Figure 3:**
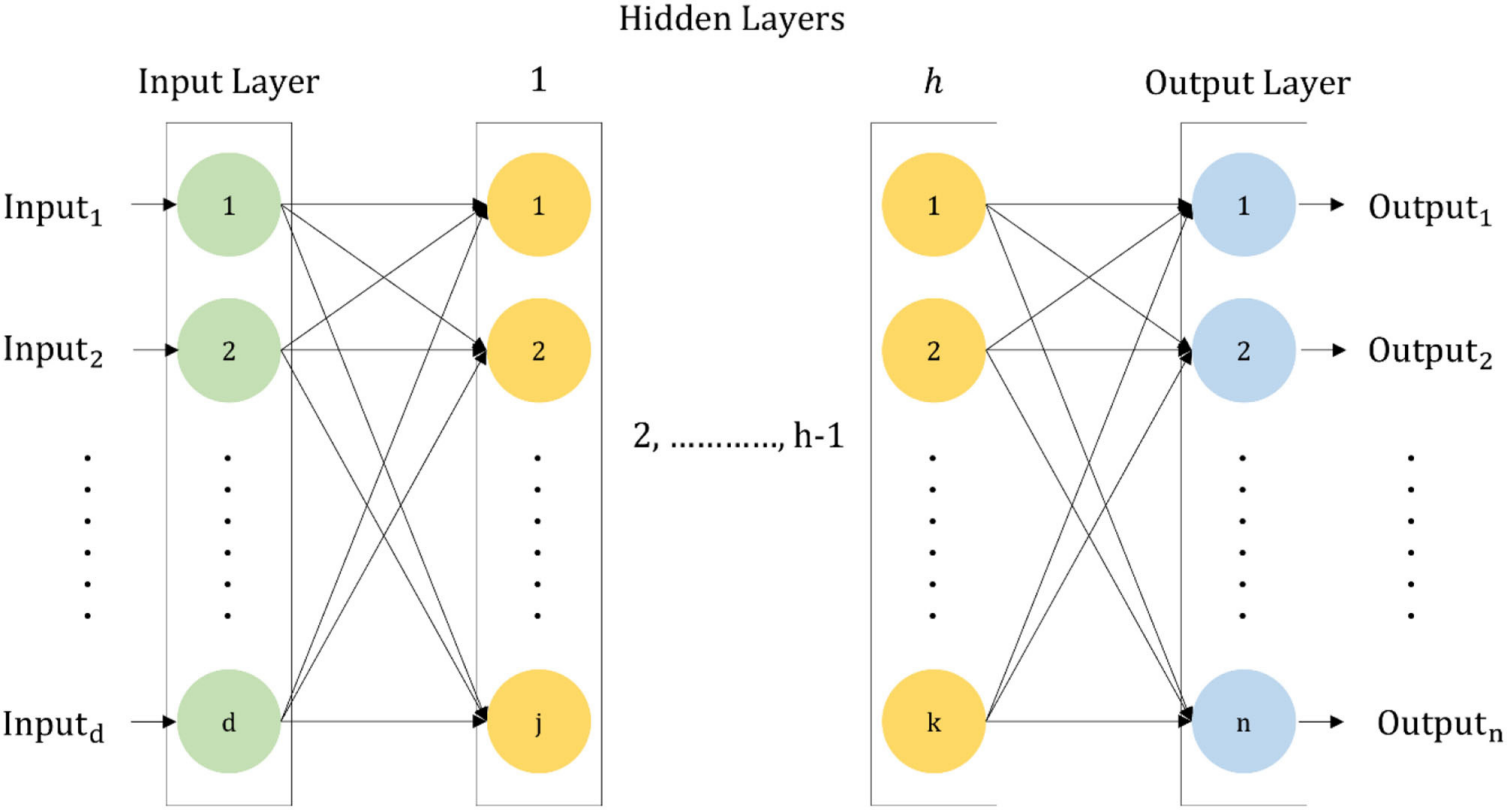
A standard structure of DNN.

A PSO-dependent algorithm (Kennedy and Eberhart, 1995) is utilized in order to select the hyperparameters of DNN. To construct the DNN, this algorithm is highly useful. A DNN is nothing but a multilayer ANN with many hidden layers (Schmidhuber, 2015). There is a full connection among the weights of DNN. The connection of every neuron in a distinct layer to all neurons of the higher end layer is situated next to it and is well established. In a feedforward manner, the propagation of the information in DNN is done (i.e.,), from the input layer to the output layer through the hidden layer. The typical DNN structure is depicted in the following Figure 3.

For different machine learning tasks, DNNs are used widely. In terms of performance, it has surpassed the ability of most of the machine learning techniques. Selecting the hyperparameter value is quite important for the performance enhancement of the DNN. For the given machine learning task, the performance of DNN is influenced by the DNN hyperparameters which help to implement it as a collection of important values that checks the behavior, architecture plan, performance accomplishment, etc. The two kinds of hyperparameters utilized are layer-based parameters and global parameters. The general behavior of DNN is defined by global parameters such as batch size, learning rate, number of layers, epochs number, and the utilized optimizer. For each layer in DNN, there is a huge dependence on layer-based parameters. Typical examples of layer-based parameters include the number of neurons, type of layer, activation function, and the initialization method. Hyperparameters can always differ from one assignment to another assignment and therefore they must be adjusted and regulated before the training process. To tune the DNN hyperparameters precisely, a machine learning expert who is specialized and updated with the current pattern recognition tasks can be utilized to overcome this problem, but the presence of such an expert is not available in most cases. The trial and error method can also be used as a potential solution to adjust these hyperparameters in a manual manner. By implementing random search/grid search, the execution of hyperparameters is done so that the search space can be handled well. On the defined attribute of hyperparameters, a grid search is executed where the identification of those specific ranges depends on the preceding proficiency of the underlying task. Then the hyperparameter values are picked up from the predetermined assortment incessantly and on the training set, the performance of DNN is evaluated. When the testing of all the amalgamation of hyperparameter values is done, the selection of the finest combination is obtained, and it is utilized to configure the DNN and enables it to evaluate it on the testing set. Grid random search and random search are almost similar to each other, but in a random search, the hyperparameter values are selected in a methodical manner and then the hyperparameter values are selected by the user from these predefined ranges in a random fashion. Bayesian optimization seems to have been a good technique for the selection of hyperparameters, however, the complex nature and huge search space criteria of the hyperparameters value of DNN make such manual algorithms exhausting. To trace the global optima of a nonlinear function, a metaheuristic algorithm/evolution is utilized which acts in a wonderful manner. To solve the DNN parametrization problem in an automatic manner, evolutionary algorithms (EA) seem to be a very versatile and promising technique. Therefore, utilizing EA for DNN hyperparameter optimization to achieve a high classification accuracy is favored.

### PSO

In a continuous search space, for the optimization of non-linear functions, the famous metaheuristic algorithm utilized is PSO (Kennedy and Eberhart, 1995). The social behavior of animals is mimicked by PSO and has a collection of many members called particles. The swarm size is nothing but the total number of particles in the swarm denoted by *S* and is an integer value. Two vectors of *N* lengths are present in each particle of the specific swarm, where the size of the dimension is expressed as *N*. In the search space of the problem, the identification of the current position of the particle is done and it is called the first vector/position vector indicated by *P*. The candidate solution to the specific problem here is nothing but the indication of the position vector. During the next iteration, in the search space of the problem, both the speed and direction of the particle are determined by the second vector termed velocity vector, which is indicated by *V*. The storing of another two vectors is quite important in the execution of PSO during every iteration. The personal best vector is the initial vector and indicated by $P_{best}^{i}$ which specifies the best position of the *i*^*th*^particle in the swarm. During every iteration, the updation of the independent personal best vector for every particle in the swarm is done. The best position traced in the swarm so far is indicated by the secondary vector known as the global best vector and is specified by *G*_*best*_. In the swarm, for all the particles, an exclusive global best vector is found, and it is updated at every iteration. The collective knowledge of the swarm is represented by the global best vector; however, the personal best vector is used to represent the cognitive knowledge of the particle. In the swarm *S*, for every particle *i* at iteration *t*, the updation of the velocity *V*and particle *P* vectors to the next iteration *t* + 1 is expressed as


(19) $$\begin{array}{l} V_{t+1}^{i}=WV_{t}^{i}+C_{1}r_{1}\left(t\right)\left(P_{best}^{i}-P_{t}^{i}\right)+C_{2}r_{2}\left(t\right)\left(G_{best}-P_{t}^{i}\right) \end{array}$$

(20) $$\begin{array}{l} P_{t+1}^{i}=P_{t}^{i}+V_{t+1}^{i} \end{array}$$

The influence of the particle velocity at the present iteration to the successive iteration is controlled by the inertia weight constant *W*. It is done so that the particle does not deviate outward to the problem search space by adjusting the particle speed and direction. The acceleration coefficients are expressed as *C*_1_ and *C*_2_ are constants. The random values are *r*_1_ and *r*_2_, and are uniformly distributed in the ranges of [0,1]. The random computation of the new values *r*_1_ and *r*_2_ is done at the start of every iteration and they are usually constant for all the swarm particles of that particular iteration. To scale both the cognitive knowledge and the collective knowledge of the particle swarm, there is a change in velocity parameter, and the constants used for this process are *C*_1_, *C*_2_, *r*_1_, *r*_2_. Thus, the optimal solution to the problem is obtained by means of new position vectors of all the particles.

### PSO for the Solution of DNN Hyperparameters

As an optimization task, the interpretation of the selection of hyperparameters of DNN is done. Minimizing the loss function *L*(*D, T*)is the primary objective, where the training set is indicated by *T*and the DNN model is indicated by *D*. The vector of the optimized hyperparameters *H*is given as an output by the PSO optimization algorithm so that the loss function is minimized after the contribution of the DNN model *D*. Then the hyperparameters *H* are utilized to trace it and then it is trained on the training set *T*. The function F^*^:*R*^*N*^ → *R* is nothing but a fitness function of the PSO-based technique, where the mapping and the training of DNN are done from a real-valued vector with a hyperparameter of length *N* to the accuracy of the DNN which has a real value. The hyperparameter vectors are used to trace it and finally on the test set *Y*, it is tested. Among all the combinations of hyperparameters, the optimal hyperparameter vector is found by the PSO algorithm so that maximum accuracy is obtained when the trained DNN is tested on the test set. The selection of hyperparameters can be any by the user to make sure that the generality of the PSO algorithm is dependent on DNN. Therefore, the hyperparameters number and the domain related to each parameter which can be set to all possible values can be well assigned by the user. The PSO-based algorithm is so efficient that all the hyperparameter vectors indicating the particles are initialized in the swarm.

For predefined parameter ranges, the validation of the proposed algorithm is quite important during execution so that the updated position and velocity vectors are authenticated. At the end of every iteration, the simultaneous checking of two various stop conditions is done so that the computations are reduced, and the convergence is made faster. The primary condition transpires when the outcome of the global best vector in terms of fitness becomes fewer than a particular threshold value ε. When the paramount number of iterations is not extended to reach the threshold value and there is no improvement in the global best vector, then this case occurs. If the paramount number of iterations are carried out well, then the second condition happens. The optimal solution *H* provides the global best vector when either the primary/secondary condition is contented and then the search process is terminated.

The four important steps are as follows:

Inputs: A training set *T*and Test set *Y*, Swarm Size *S*, Acceleration constant *C*_1_, *C*_2_, Number of hyperparameters *N*, evolution threshold parameter(ε), Inertia constant *W*, paramount number of iterations (*t*_max_), minimum and maximum velocity value (*V*_min_) and (*V*_max_). The final optimal solution is given as *H*

Step 1: Preprocessing Phase:

a) The process is initialized
b) The inputs *N, S, V*_min_, *V*_max_ are given
c) The domains for *h*^*k*^, *k* ← 1 to *N*are defined.
d) The hyperparameters and velocity generators are created
e) The *P* and *V*vectors of *S* particles are initialized each of *N* length.

Step 2: Initialize Phase:

a) Input *T, Y*, ε, *C*_1_, *C*_2_, *W, t*_max_
b) $P_{best}^{i}\leftarrow -\infty ,i\leftarrow \;1toS$
c) *G*_*best*_ ← −∞
d) For all *S*particles, F*(*P*) is computed and then $P_{best}^{i}$ is updated.
e) *G*_*best*_ is updated
f) *t* ← 1

Step 3: Evolution Phase:

a) *r*_1_(*t*) and *r*_2_(*t*) are computed
b) V, P, F*(*P*)and $P_{best}^{i}$ for all the *S*particles are computed
c) *G*_*best*_ is updated
d) *t* ← *t* + 1
e) Check for stop conditions. If yes, proceed to the finish stage and if no, repeat the evolution stage

Step 4: Finishing Phase:

a) *H* ← *G*_*best*_ is given as output
b) Terminated

### Parameters of PSO

The PSO parameter (*S, C*_1_, *C*_2_, *W, V*_max_, *V*_min_, *t*_max_, ε)selection is quite a complex process. Previous studies have recommended quite a lot of values of the PSO parameters that could be utilized. The value ranges for every PSO parameter are shown in Table 3. For each parameter, a value is selected randomly and during the PSO execution, it is fixed as a constant.

**Table 3:** PSO parameters values/ranges.

**Parameter**	**Value/Ranges**
*C*_1_	0 to 4
*C*_2_	0 to 4
*S*	[5, 75]
*V*_min_	0
*V*_max_	1
W	[0.5, 0.8]
*t*_max_	[20, 120]
ε	[0.1, 0.00001]

### Implementation of Swarm DNN

The description of the proposed Swarm DNN in text classification is as follows. The proposed methodology comprises four successive stages such as (a) Initialization Stage, (b) Optimization Stage, (c) Extraction of meaningful results Stage, and finally (d) Termination Stage. All the necessary operating parameters are initialized, and the text input files are prepared well. The text comprises the training set which is followed and accompanied by the test set. The parameters for PSO elements in DNN experiments are set as described in the four cases. The experiment was repeated for hundreds of cases by trial-and-error method and finally, four cases that gave the highest classification accuracy results were considered and analyzed in this work.

Case 1:


$$\begin{array}{l} \;S=15,V_{\min}=0,V_{\min}=1,t_{\max}=60,\varepsilon =10^{-2},\\ C_{1}=C_{2}=1,W=0.5 \end{array}$$

Case 2:


$$\begin{array}{l} S=30,V_{\min}=0,\;V_{\min}=1,t_{\max}=80,\\ \varepsilon =10^{-3},C_{1}=C_{2}=1,W=0.6 \end{array}$$

Case 3:


$$\begin{array}{l} S=50,V_{\min}=0,\;V_{\min}=1,t_{\max}=100,\\ \varepsilon =10^{-4},C_{1}=C_{2}=2,W=0.7 \end{array}$$

Case 4:


$$\begin{array}{l} S=65,\;V_{\min}=1,t_{\max}=120,\\ \varepsilon =10^{-4},C_{1}=C_{2}=2,W=0.8 \end{array}$$

Thus, the DNN hyperparameters and their domains are defined in this initialization stage. Twelve various DNN hyperparameters *N* = 12 are utilized. The hyperparameters of DNN and its respective domain along with the explanation are shown in Table 4.

**Table 4:** DNN hyperparameter and its domain.

**Hyperparameters**	**Domain**	**Explanation**
Momentum	[0.1, 0.9]	Continuous
Learning Rate	[0.1, 0.9]	Continuous
Drop rate	[0.1, 0.9]	Continuous
Delay	[0.0001, 0.01]	Continuous
Number of hidden layers	[1, 10]	Discrete with step = 1
Number of neurons in hidden layer	[1, 300]	Discrete with step = 1
Number of epochs	[5, 25]	Discrete with step = 5
Batch size	[100, 1,000]	Discrete with step = 100
Layer type	[1, 2]	Discrete with step = 1
Optimizer	[1, 6]	Discrete with step = 1
Initialization function	[1, 8]	Discrete with step = 1
Activation function	[1, 8]	Discrete with step = 1

**Figure 4:**
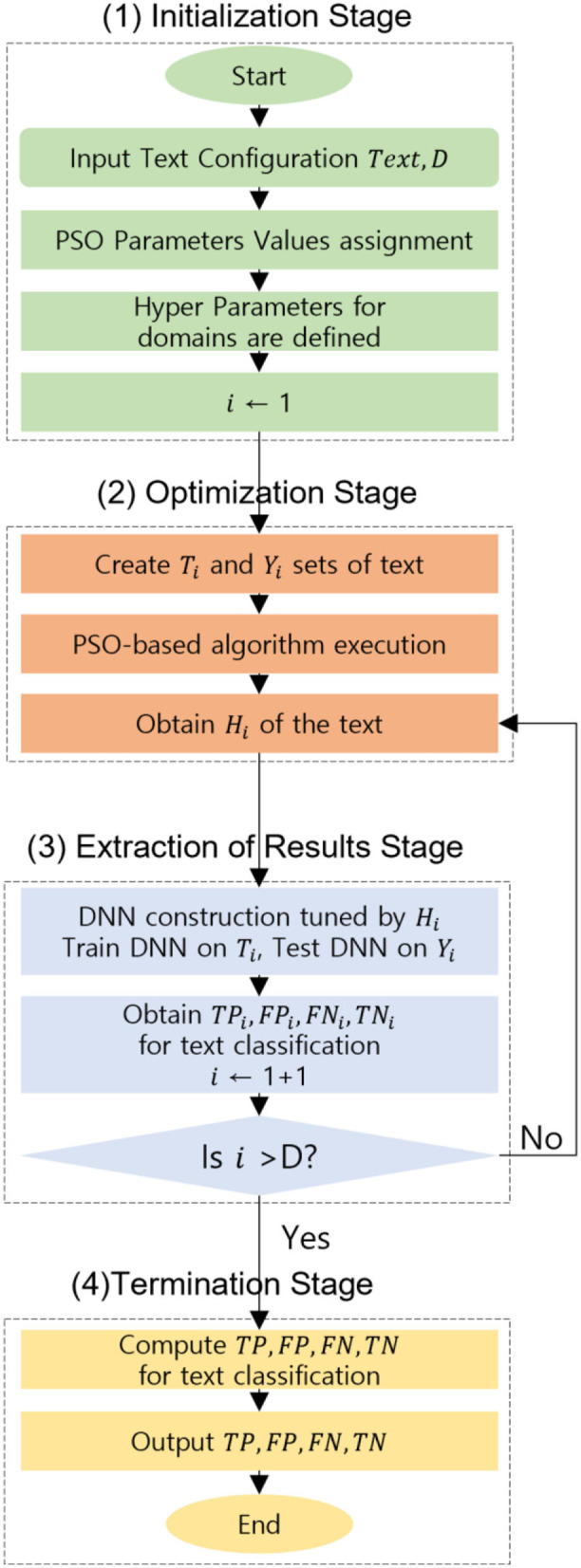
Flow chart of the proposed Swarm DNN.

Most of the hyperparameters utilized are numerical with an exception to layer type, activation function, and initialization for an optimizer as they are categorical. The indexing of all the feasible values to a successive number of ranges from one to that specific length of the list is done. Adagrad, Adam, Adamax, Nadam, RMS prop, and SGD are the elements included in the optimizer list. Dropout and Dense are the two elements present in the layer type. The elements of the initialization function include Normal, Uniform, He normal, He uniform, Zero, Glorot uniform, Glorot normal, and Leun uniform. Eight elements are present in the activation list such as linear, sigmoid, hard_sigmoid, Softmax, ReLU, Tanh, Soft plus, and Soft sign. The elements belonging to all the categorical hyperparameters are mentioned and explained in Keras execution. For every text, the optimization and result extraction stages will be implemented. The splitting of the data into two independent sets training and test sets *T*_*i*_ and *Y*_*i*_ are done by the optimization stage. From text to numerical values, the conversion of the training and test sets are performed and finally, they are normalized in [0,1]. Then to the PSO-based algorithm, these sets are given as input so that the optimized hyperparameters vector *H*_*i*_ are found. The construction of the DNN tuned *H*_*i*_ is done by the results extraction stage, then the DNN is trained on *T*_*i*_ and tested *Y*_*i*_. The classification output values are extracted and processed. The execution of the finishing stage is done when all the text in the data is completed. The outcomes are obtained at the termination stage and at the end of the DNN experiment. The flow chart is explained in Figure 4.

## Results and Discussion

In this section, the evaluation criteria for text classification, the dataset explanation, and the results of the proposed deep learning architectures, followed by a comparison with other works are elaborated in detail.

### Evaluation Criteria for Text Classification Results

The accuracy is mainly used as a classification criterion for the text classification results. In the fields of statistical classification, to evaluate its performance of it, this index is widely used.

The performance of the classifier is reflected by accuracy and is expressed as:


(21) $$\begin{array}{l} Accuracy=\frac{TP+TN}{TP+TN+FP+FN} \end{array}$$

The recall is expressed as:


(22) $$\begin{array}{l} \text{Recall}=\frac{TP}{TP+FN} \end{array}$$

The Precision is expressed as:


(23) $$\begin{array}{l} \text{Precision}=\frac{TP}{TP+FP} \end{array}$$

The geometric mean (g-mean) is expressed as follows:


(24) $$\begin{array}{l} gmean=\sqrt{\frac{TP\times TN}{\left(TP+FN\right)\times \left(TN+FP\right)}} \end{array}$$

The Mathews Correlation Coefficient (MCC) is expressed as follows:


(25) $$\begin{array}{l} MCC=\frac{\left(TP\times TN\right)-\left(FP\times FN\right)}{\sqrt{\left(TP+FN\right)\times \left(TP+FP\right)\times \left(TN+FP\right)\times \left(TN+FN\right)}} \end{array}$$

### Dataset Explanation

The methodology is tested on two datasets that are considered in our experiments, 20 newsgroups, and BBC news datasets. There are 20 different news comment groups in the 20 newsgroups dataset where each group indicates a news topic. Three different versions are present on the website (http://qwone.com/~jason/20Newsgroups/). The second version is selected where a total of 18,846 documents are found. The division of the dataset is done into two main parts, where for the train set, 11,314 documents are found and for the test set, 7,532 documents are found. The BBC news dataset consists of many news documents from the BBC website (http://www.bbc.co.uk/news/business/market_data/overview/). A total of 2,225 documents are included in the dataset which corresponds to five topics such as technology, business, politics, sports, and entertainment. For the train set, 1,600 documents are selected randomly and for the test set, 625 documents are selected.

### Results of the Proposed ECCNN and Swarm DNN Architectures

The effect of various parameters of the DE algorithm with the CCNN architecture is analyzed, such as population size, the main implementation procedure of DE, such as DE/best/1 or DE/best/2, mutation parameters *F*, crossover probability, and the results are shown in Tables 5–**10**. With the assignment of random values for more than a hundred possible combinations, finally, the six best results of the ECCNN combination are shortlisted and reported in Tables 5–**10**.

**Table 5:** Results of the ECCNN architecture with DE/best/1 and DE/best/2 strategy, F = 0.2, CR = 0.3.

**DE parameters**	**DE/best/1**	**DE/best/1**	**DE/best/2**	**DE/best/2**
	Population size 60	Population size 90	Population size 60	Population size 90
Dataset utilized	Accuracy	Accuracy	Accuracy	Accuracy
20 newsgroup	72.34	78.91	73.24	79.22
BBC newsgroup	87.63	82.35	81.56	89.12

Table 5 explains the results of the ECCNN architecture with DE/best/1 and DE/best/2 strategy with population sizes of sixty and ninety along with F and CR set as 0.2 and 0.3, respectively. Under such a case, a classification accuracy of 79.22% for 20 newsgroup datasets for the DE/best/2 strategy and the second-highest classification accuracy of 78.91% with the DE/best/1 strategy was obtained again for 20 newsgroup datasets. For the BBC newsgroup dataset, a classification accuracy of 89.12% with DE/best/2 strategy and the second-highest classification accuracy of 87.63% with DE/best/1 strategy was obtained. Table 6 explains the results of the CNN architecture with DE/best/1 and DE/best/2 strategy with population sizes of sixty and ninety along with F and CR set as 0.4 and 0.5, respectively. Under this case, the overall highest classification accuracy of 97.11% was obtained for the BBC newsgroup dataset with the DE/best/2 strategy, and overall high accuracy of 88.76% was obtained for the 20 newsgroup dataset with DE/best/2 strategy. Table 7 explains the results of the ECCNN architecture with DE/best/1 and DE/best/2 strategy with population sizes of 60 and 90 along with F and CR set as 0.6 and 0.8, respectively. Under this case, a classification accuracy of 80.34% for the DE/best/2 strategy with a 20-newsgroup dataset was obtained and an accuracy of 91.37% with the DE/best/2 strategy with a BBC newsgroup dataset was obtained. Table 8 explains the results of the ECCNN architecture with DE/best/1 and DE/best/2 strategy with population sizes of 60 and 90 along with F and CR set as 0.8 and 0.2, respectively. Under this case, a classification accuracy of 89.46% for DE/best/1 strategy with BBC newsgroup dataset was obtained and an accuracy of 84.76% for DE/best/2 strategy with 20 newsgroup dataset was obtained. Table 9 explains the results of the ECCNN architecture with DE/best/1 and DE/best/2 strategy with population sizes of sixty and ninety along with F and CR set as 0.6 and 0.4, respectively. Under this case, a classification accuracy of 94.25% for DE/best/1 strategy with BBC newsgroup dataset was obtained and an accuracy of 82.36% for DE/best/1 strategy with 20 newsgroup dataset was obtained.

**Table 6:** Results of the ECCNN architecture with DE/best/1 and DE/best/2 strategy, F = 0.4, CR = 0.5.

**DE Parameters**	**DE/best/1**	**DE/best/1**	**DE/best/2**	**DE/best/2**
	Population size 60	Population size 90	Population size 60	Population size 90
Dataset utilized	Accuracy	Accuracy	Accuracy	Accuracy
20 newsgroup	81.62	84.45	85.56	88.76
BBC newsgroup	92.35	92.49	96.12	97.11

**Table 7:** Results of the ECCNN architecture with DE/best/1 and DE/best/2 strategy, F = 0.6, CR = 0.8.

**DE parameters**	**DE/best/1**	**DE/best/1**	**DE/best/2**	**DE/best/2**
	Population size 60	Population size 90	Population size 60	Population size 90
Dataset utilized	Accuracy	Accuracy	Accuracy	Accuracy
20 newsgroup	79.12	77.45	80.34	78.35
BBC newsgroup	89.23	88.35	89.24	91.37

**Table 8:** Results of the ECCNN architecture with DE/best/1 and DE/best/2 strategy, F = 0.8, CR =0.2.

**DE parameters**	**DE/best/1**	**DE/best/1**	**DE/best/2**	**DE/best/2**
	Population size 60	Population size 90	Population size 60	Population size 90
Dataset utilized	Accuracy	Accuracy	Accuracy	Accuracy
20 newsgroup	78.35	79.35	84.76	82.34
BBC newsgroup	88.45	89.46	85.56	85.13

**Table 9:** Results of the ECCNN architecture with DE/best/1 and DE/best/2 strategy, F = 0.6, CR = 0.4.

**DE parameters**	**DE/best/1**	**DE/best/1**	**DE/best/2**	**DE/best/2**
	Population size 60	Population size 90	Population size 60	Population size 90
Dataset utilized	Accuracy	Accuracy	Accuracy	Accuracy
20 newsgroup	80.23	82.36	79.23	78.23
BBC newsgroup	91.04	94.25	89.94	92.46

Table 10 explains the results of the ECCNN architecture with DE/best/1 and DE/best/2 strategy with population sizes of 60 and 90 along with F and CR set as 0.5 and 0.3, respectively. Under this case, a classification accuracy of 81.24% with DE/best/2 strategy with 20 newsgroup datasets was obtained and high accuracy of 91.48% for DE/best/1 strategy with BBC newsgroup dataset was obtained. Table 11 shows the results of the Swarm DNN architecture for the parameters of the four different cases of values the highest classification accuracy of 97.32% was obtained for Case 4 parameter values with the BBC news dataset and a high classification accuracy of 87.99% was obtained for Case 3 parameter values with 20 newsgroup datasets. The final comparison of the best results for the two developed deep learning models implemented for the two datasets is computed and tabulated in Table 12.

**Table 10:** Results of the ECCNN architecture with DE/best/1 and DE/best/2 strategy, F = 0.5, CR = 0.3.

**DE parameters**	**DE/best/1**	**DE/best/1**	**DE/best/2**	**DE/best/2**
	Population size 60	Population size 90	Population size 60	Population size 90
Dataset utilized	Accuracy	Accuracy	Accuracy	Accuracy
20 newsgroup	79.45	80.21	78.25	81.24
BBC newsgroup	91.48	89.24	84.59	87.31

**Table 11:** Results of the Swarm DNN architecture for the parameters of the four different cases.

**PSO parameters**	**PSO Parameters: Case 1**	**PSO Parameters: Case 2**	**PSO Parameters: Case 3**	**PSO Parameters: Case 4**
Dataset utilized	Accuracy	Accuracy	Accuracy	Accuracy
20 newsgroup	84.45	82.21	87.99	85.24
BBC newsgroup	93.48	93.24	95.59	97.32

**Table 12:** Consolidated result analysis of the proposed techniques in text classification.

**Dataset**	**Model**	**Accuracy**	**Precision**	**Recall**	**Geometric mean**	**MCC**
20 newsgroup	ECCNN	88.76	82.45	81.35	81.23	79.45
	Swarm DNN	87.99	83.13	82.45	82.06	80.13
BBC	ECCNN	97.11	91.36	90.43	89.13	88.31
newsgroup	Swarm DNN	97.32	90.18	89.48	88.12	87.06

From Table 12, it is understood that when the proposed ECCNN model is implemented with the DE/best/2 strategy and with an F and CR values of 0.4 and 0.5 respectively, a classification accuracy of 88.76%, precision of 82.45%, Recall of 81.35%, the geometric mean of 81.23% along with an MCC of 79.45% is obtained for the 20 Newsgroup dataset. Similarly, when the proposed ECCNN model is implemented with the DE/best/2 strategy and with an F and CR values of 0.4 and 0.5 respectively, a classification accuracy of 97.11%, precision of 91.36%, Recall of 90.43%, Geometric mean of 89.13% along with an MCC of 88.31% is obtained for the BBC Newsgroup dataset.

Similarly, when the proposed Swarm DNN model is implemented with PSO parameters pertaining to case 4, a classification accuracy of 87.99%, precision of 83.13%, Recall of 82.45%, and Geometric mean of 82.06% along with an MCC of 80.13% is obtained for the 20 Newsgroup dataset. Similarly, when the proposed Swarm DNN is implemented with PSO parameters pertaining to case 4, a classification accuracy of 97.32%, precision of 90.18%, Recall of 89.48%, and Geometric mean of 88.12% along with an MCC of 87.06% is obtained for the BBC Newsgroup dataset. As far as statistical tests are concerned, initially, a 2-sided Wilcoxon rank-sum test was conducted and the ρ value obtained was <0.05, thereby obtaining a high confidence level. A Kruskal Wallis test too was analyzed and the value ρ obtained was <0.01 proving its statistical significance and correctness. A Friedman test too was conducted to analyze the unique differences between multiple algorithms of the datasets and upon testing there were clear and distinct variations. Finally, a Cohen's Kappa coefficient test too was conducted, and the values always reached a good agreement or a very good agreement category.

### Comparison With Other Works Utilizing the Same Database

Some of the commonly used databases for text classification include Standard Reuters 21578, MNIST dataset, MIT newspaper, Wikipedia XML Corpus, MTI ML Site, Jeeves Support System database, GENIA and CRAFT, Nature Database, Dream bank Report Corpus, along with the databases used in this paper such as 20 newsgroups and BBC newsdata (Altinel and Ganiz, 2018). Very few peer-reviewed works have been reported in high-quality journals and literature with the 20 newsgroups and BBC newsdata and some important works are considered here for comparison with the results obtained in this work. A vigorous text classifier dependent on a Denoising Deep Neural Network (DDNN) was proposed by Aziguli and they reported a classification accuracy of 92.86% for the BBC newsgroup dataset and 73.78% for the 20-newsgroup dataset (Aziguli et al., 2017). A multilayer classification was utilized by Pradhan et al. reporting a classification accuracy of 97.67% for BBC news and 86.70% for 20 newsgroup datasets (Pradhan et al., 2017). An Instance-Infused LSTM was proposed by Chowdhury et al. reporting an accuracy of 78.29% for the News 20 dataset and 96.09% for the BBC news dataset (Chowdhury et al., 2019). A combination of CNN with Long Short-Term Memory (LSTM) was utilized by Camacho et al. reporting 97% accuracy for the BBC news group set and 90.7% for the 20 newsgroup sets (Camacho-Collados and Pilehvar, 2017). A Deep Belief Network (DBN) with Softmax model (Jiang et al., 2018), deep learning with meta-thesaurus (Liu et al., 2017), and LSTM (Shih et al., 2017) were utilized giving a classification accuracy of 85.57%, 69.82%, and 86.2% for the 20-newsgroup dataset, respectively. Support Vector Machine (SVM) and Naïve Bayesian Classifier (NBC) were utilized by Shirsat reporting a classification accuracy of 96.46% with SVM and 94.16% with NBC for the BBC news dataset (Shirsat et al., 2019). A bigram alphabet approach for text representation and classification of the BBC news dataset was reported by Elghannam reporting a classification accuracy of 92.6% (Elghannam, 2019). However, in our work, we introduced swarm intelligence to the modified deep learning models and developed interesting results. By and large, the work produced a higher classification accuracy of 97.32% using the Swarm DNN model and 97.11% using the ECCNN model for BBC datasets, respectively. Similarly, this work reports a high classification accuracy of 87.99% using the Swarm DNN model and 88.76% using the ECCNN model for 20-news group datasets respectively, which is quite a commendable performance when compared to other works. As the methods proposed in this work are quite interesting and easy to implement, these models can be very well applied to other datasets too.

## Conclusion and Future Work

In the area of NLP, text classification has been a very interesting issue. For big data analysis, good classification accuracy is very important to implement NLP for scientific data analytics. To manage a huge amount of text documents in the fields of web mining, information retrieval, natural language processing, and content security research areas, text classification plays a huge role. The assignment of one or more predefined classes to a natural text document based on the knowledge obtained from text expression is the vital task of text classification. Therefore, the development of effective and versatile algorithms is quite challenging in the area of text classification because of the large size of the text data. In this work, two successful deep learning models such as ECCNN and Swarm DNN were developed and tested on two datasets and the results were obtained. A very high classification accuracy of 97.32% was obtained for the BBC newsgroup dataset when utilized with the Swarm DNN model. The second-highest classification accuracy of 97.11% was obtained for the BBC newsgroup dataset with the ECCNN model, where the DE strategy was DE/best/2 and with a specific set of assigned mutation scaling and crossover probability parameters. The third-highest classification accuracy rate of 87.99% was obtained for the 20-newsgroup dataset when the Swarm DNN model was utilized. The fourth-highest classification accuracy of 88.76% was obtained for the 20-newsgroup dataset when the ECCNN model was utilized. Future works aim to work with many other nature-inspired and ensemble deep learning models for efficient text classification purposes.

## Data Availability Statement

All the relevant programming codes implemented in the work can be obtained from the corresponding author upon request.

## Author Contributions

All authors listed have made a substantial, direct, and intellectual contribution to the work and approved it for publication.

## Funding

This work was supported by the Institute of Information & Communications Technology Planning & Evaluation (IITP) grant funded by the Korea government (MSIT) (No. 2021-0-02068, Artificial Intelligence Innovation Hub) and partly supported and funded by the Korean National Police Agency (Project Name: AI-based Crime Investigation Support System / Project Number: PR10-02-000-21).

## Conflict of Interest

The authors declare that the research was conducted in the absence of any commercial or financial relationships that could be construed as a potential conflict of interest.

## Publisher's Note

All claims expressed in this article are solely those of the authors and do not necessarily represent those of their affiliated organizations, or those of the publisher, the editors and the reviewers. Any product that may be evaluated in this article, or claim that may be made by its manufacturer, is not guaranteed or endorsed by the publisher.
